# Moderate intensity supine exercise causes decreased cardiac volumes and increased outer volume variations: a cardiovascular magnetic resonance study

**DOI:** 10.1186/1532-429X-15-96

**Published:** 2013-10-24

**Authors:** Katarina Steding-Ehrenborg, Robert Jablonowski, Per M Arvidsson, Marcus Carlsson, Bengt Saltin, Håkan Arheden

**Affiliations:** 1 Copenhagen Muscle Research Centre, Copenhagen, Denmark; 2 Danish Research Centre for Magnetic Resonance, Hvidovre Hospital, Copenhagen, Denmark; 3 Department of Clinical Physiology, Lund University, Lund University Hospital Lund, Lund, Sweden

**Keywords:** Physiology, Total heart volume variation, Ventricle, Cardiac pumping, Cardiovascular magnetic resonance

## Abstract

**Background:**

The effects on left and right ventricular (LV, RV) volumes during physical exercise remains controversial. Furthermore, no previous study has investigated the effects of exercise on longitudinal contribution to stroke volume (SV) and the outer volume variation of the heart. The aim of this study was to determine if LV, RV and total heart volumes (THV) as well as cardiac pumping mechanisms change during physical exercise compared to rest using cardiovascular magnetic resonance (CMR).

**Methods:**

26 healthy volunteers (6 women) underwent CMR at rest and exercise. Exercise was performed using a custom built ergometer for one-legged exercise in the supine position during breath hold imaging. Cardiac volumes and atrio-ventricular plane displacement were determined. Heart rate (HR) was obtained from ECG.

**Results:**

HR increased during exercise from 60±2 to 94±2 bpm, (p<0.001). LVEDV remained unchanged (p=0.81) and LVESV decreased with −9±18% (p<0.05) causing LVSV to increase with 8±3% (p<0.05). RVEDV and RVESV decreased by −7±10% and −24±14% respectively, (p<0.001) and RVSV increased 5±17% during exercise although not statistically significant (p=0.18). Longitudinal contribution to RVSV decreased during exercise by −6±15% (p<0.05) but was unchanged for LVSV (p=0.74). THV decreased during exercise by −4±1%, (p<0.01) and total heart volume variation (THVV) increased during exercise from 5.9±0.5% to 9.7±0.6% (p<0.001).

**Conclusions:**

Cardiac volumes and function are significantly altered during supine physical exercise. THV becomes significantly smaller due to decreases in RVEDV whilst LVEDV remains unchanged. THVV and consequently radial pumping increases during exercise which may improve diastolic suction during the rapid filling phase.

## Background

Total heart volume at rest has a strong correlation to peak exercise capacity in healthy normal subjects and athletes [1,2]. When going from rest to exercise the normal heart in a sedentary individual can increase its cardiac output from 5 L/min to 20–25 L/min [3]. This change has been attributed to an increase in heart rate and stroke volume. In turn, the stroke volume can increase either by an increase in end-diastolic volume (EDV), decrease in end-systolic volume (ESV), or both. The effects on left ventricular (LV) volumes during physical exercise remain controversial. Previous studies using radionuclide angiography or echocardiography have shown both unchanged and increased LV end-diastolic volumes (LVEDV) during upright and supine exercise compared to resting values in the same position [4-12]. Although most studies show a decrease in ESV during exercise, Sundstedt et al. [12] showed unchanged ESV during supine exercise using echocardiography. Few studies have investigated the effects of exercise on the right ventricle [13,14] and further studies are needed to understand how physical exercise affects left and right cardiac volumes and subsequently the stroke volume (SV).

Ventricular stroke volume is ejected by a combination of longitudinal and radial contraction of the ventricle [15-18]. At rest the longitudinal contribution to SV has been shown to be 60% for the LV and 80% for the RV and radial contribution is 40% and 20% respectively [15,17,19]. It has been shown that during exercise there is a significant increase in the mitral valve displacement during exercise [20]. Longitudinal pumping is calculated as the atrio-ventricular plane displacement (AVPD) multiplied by the short-axis area of the ventricle and an increase in AVPD may therefore affect the longitudinal contribution to SV. Several studies have suggested that at higher heart rates a larger longitudinal contribution may keep the outer volume variation of the heart to a minimum rendering less energy to be wasted on moving surrounding tissues [21-23]. However, this remains to be explored.

Therefore, the aim of this study was to determine left and right ventricular volumes, left and right atrial volumes and total heart volumes as well as longitudinal and radial pumping during rest and physical exercise using cardiovascular magnetic resonance (CMR).

## Methods

This study was approved by the Regional Ethical Review Board in Lund, Sweden and follows the Declaration of Helsinki. All participants provided written informed consent. All CMR examinations were performed at Skane University Hospital Lund, Sweden.

### Study population and experimental setup

Twenty-six healthy volunteers (six women) aged 30±8 years (mean±SD) (range 19–59) underwent CMR at rest and during exercise with one-legged knee extensions. A custom built MR-compatible ergometer provided concentric resistance during knee extension by a rope and pulley system which was integrated with a mechanically braked flywheel. A strap connected to a variable weight system provided resistance and weight was added to achieve an exercise level at approximately 40 beats per minute (bpm) higher than the subjects’ resting heart rate. The subjects were connected at the ankle to the axle of the flywheel by a rope and the extension phase of the exercise turned the flywheel. Gravity returned the leg to the starting position and a gearing system on the axle returned the rope to the starting position at the end of each duty cycle.

### Reproducibility of exercise measurements

Six subjects underwent a total of five CMR scans to investigate the reproducibility of volumetric measurements during exercise and the potential effects of different respiratory phases as well as differences in exercising muscle mass. The scans were divided into two sessions with a 1.5 hour rest outside the scanner between them. Session 1 included a) CMR at rest; b) CMR with 1-legged exercise at end-expiratory breath hold; and c) CMR with 2-legged exercise at end-expiratory breath hold. Session 2 included a) CMR with 1-legged exercise at end-expiratory breath hold; and b) CMR with 1-legged exercise at end-inspiratory breath hold with the instructions to keep an open glottis and avoid Valsalva-like increases in intra-thoracic pressures.

### Cardiac magnetic resonance imaging

A 1.5T scanner (Philips Achieva, Philips, Best, The Netherlands) with a 5 channel cardiac coil was used to scan all subjects in the supine position. A balanced steady-state free-precession (bSSFP) sequence with retrospective ECG gating was used to acquire images of the heart (repetition time typically 3.0 ms, turbo factor 16, echo time 1.5 ms, flip angle 60°, reconstructed to a spatial resolution of 1.4 × 1.4 mm, acquired temporal resolution typically 50 ms reconstructed to 30 ms, and slice thickness 8 mm with no slice gap). After defining the long-axis orientation of the heart, short-axis images covering the heart from the base of the atria to the apex of the ventricles were obtained. Breath-hold during imaging during exercise was typically 6 s for long-axis images and 8–10 s for each short-axis slice. An ECG-triggered phase-contrast sequence was used to measure blood flow in the aorta (repetition time 8.6 ms, echo time 6.4 ms, 150 cm/s velocity encoding, slice thickness 8 mm). The measurement plane was positioned perpendicular to the vessel. Heart rate was obtained from the ECG during image acquisition.

### Atrial and ventricular volumes

All measurements were done using the software Segment 1.8 (http://segment.heiberg.se) [24]. Left and right atrial volumes were measured in short-axis images at the time of ventricular end-diastole and ventricular end-systole. Left ventricular mass (LVM), end-diastolic volume (LVEDV), end-systolic volume (LVESV) and stroke volume (LVSV) were measured in short-axis images using planimetry, by manual delineation of endocardial and epicardial borders of the left ventricle. Papillary muscles were not included in LVM measurements. Right ventricular end-diastolic volume (RVEDV), end-systolic volume (RVESV) and stroke volume (RVSV) were measured in short-axis images by manual delineation of the right ventricular endocardial and epicardial border.

Total heart volume (THV) was measured in short-axis images by planimetry [22] and was defined as the volume of all structures within the pericardium, including myocardium, blood pool, atria, pericardial fluid and the proximal parts of the great vessels.

### Ventricular pumping

Atrio-ventricular plane displacement (AVPD) was determined from CMR long-axis images as previously described [15]. Longitudinal pumping of the left and right ventricle was calculated as the distance travelled by the AV-plane multiplied by epicardial short-axis area of the ventricle [25]. Radial pumping was determined from the total heart volume variation (THVV) [15]. Longitudinal and radial contribution to SV (%) was calculated as longitudinal pumping divided by SV and radial pumping divided by SV.

### Statistical analysis

Statistical analysis was performed using SPSS statistics 20 (IBM, Chicago, IL, USA) and a p-value <0.05 was considered statistically significant. Paired t-tests were used to test for changes between rest and exercise. Wilcoxon non-parametric test was used to test for differences between rest and exercise in the subgroup of six subjects who underwent a total of five scans to investigate the reproducibility of measurements. Results are presented as mean ±SEM unless stated otherwise. Inter-observer variability was determined for the left ventricular measurements in ten subjects during rest and exercise.

## Results

**Figure 1:**
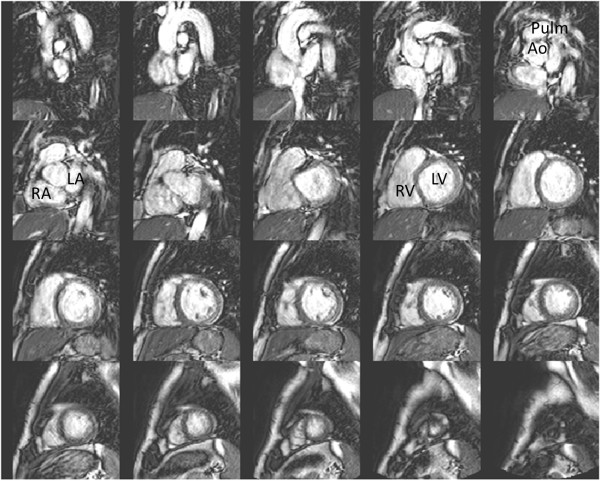
**Short**-**axis images showing the typical image quality during exercise.** These images were acquired at a heart rate of 119 bpm. The top left image shows the most basal short-axis slice showing the roof of the atria and the bottom right image shows the most apical slice of the ventricles. Ao – aorta, LA –left atrium, LV – left ventricle, Pulm – pulmonary trunk, RA – right atrium, RV – right ventricle.

Subject characteristics are presented in Table 1. All subjects reported to be healthy and none of the subjects showed any signs of cardiac disease on the CMR scan. In three subjects the same short-axis slice was imaged twice due to difficulties in breath holding during exercise. These extra slices were identified and removed before the images were analysed. Figure 1 and Additional files 1, 2, 3 and 4 show typical examples of the image quality during exercise.

**Table 1:** **Subject characteristics and cardiac volumes at rest for men and women** (**mean**±**SD**)

	**Men n=20**	**Women n=6**
**Age (years)**	30±9	29±8
**Weight (kg)**	78±12	61±14
**Height (m)**	1.80±0.07	1.68±0.07
**THV (mL)**	861±145	586±123
**LVEDV (mL)**	197±34	148±36
**RVEDV (mL)**	219±37	149±39
**LVSV (mL)**	109±19	87±22
**RVSV (mL)**	110±17	84±23
**LVM (g)**	126±22	79±17
**LAes (mL)**	89±19	65±24
**RAes (mL)**	134±33	90±22

g = gram, kg = kilogram, LAes = left atrial volume measured at ventricular end-systole, LVEDV = left ventricular end-diastolic volume, LVM = left ventricular mass, LVSV = left ventricular stroke volume, m = metre, mL = millilitre, RAes = right atrial volume measured at ventricular end-systole, RVEDV = right ventricular end-diastolic volume, RVSV = right ventricular stroke volume, THV = total heart volume, THVV = total heart volume variation.

### Heart rate and cardiac volumes

Heart rate increased significantly during exercise from 60±2 to 94±2 bpm (p<0.001). Left atrial volumes at end-diastole decreased from 39±3 to 35±3 mL (p<0.05) as did right atrial end-diastolic volumes from 65±5 to 56±4 mL (p<0.05). At ventricular end-systole, where the atria reach their largest volumes, left atrial volumes were unchanged (84±4 and 85±5 mL, p=0.72) and right atrial volume decreased significantly from 124±7 to 103±7 mL (p<0.001) with exercise.

**Figure 2:**
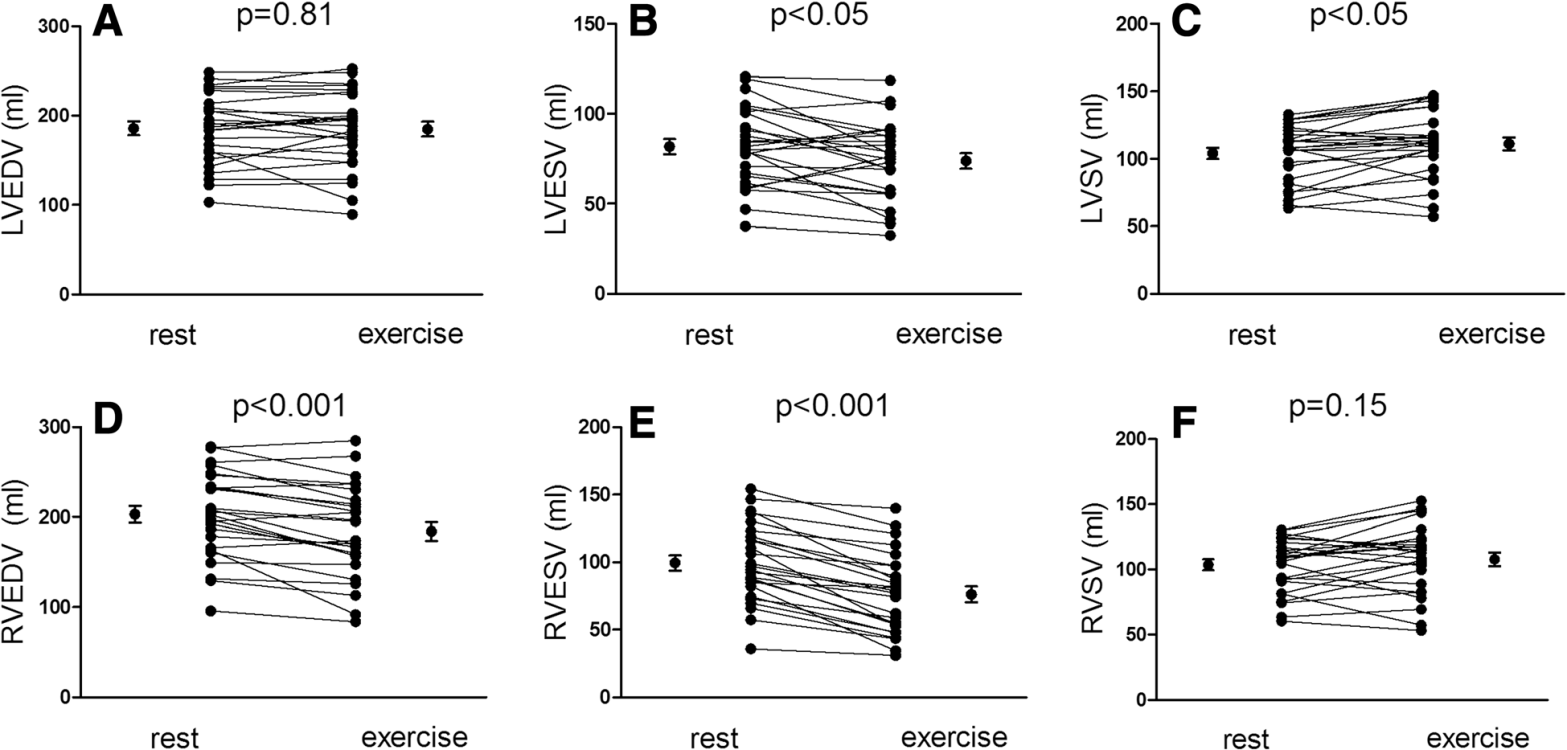
**Left and right ventricular volumes and stroke volumes at rest and exercise.** Upper panel shows no changes in left ventricular end-diastolic volumes **(A)** and a small but significant decrease in end-systolic volume **(B)**, leading to an increased stroke volume **(C)**. Lower panel show a significant decrease in right ventricular end-diastolic volume **(D)** and end-systolic volume **(E)**. Right ventricular stroke volume increased during exercise, however not statistically significant **(F)**. Error bars denote mean and standard error of the mean (SEM).

**Figure 3:**
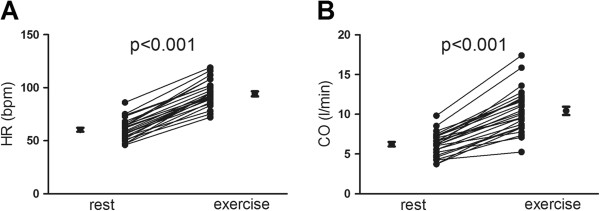
**Heart rate and cardiac output at rest and exercise.** Heart rate **(A)** and cardiac output **(B)** increased significantly from rest to exercise. Error bars denote mean and standard error of the mean (SEM).

**Figure 4:**
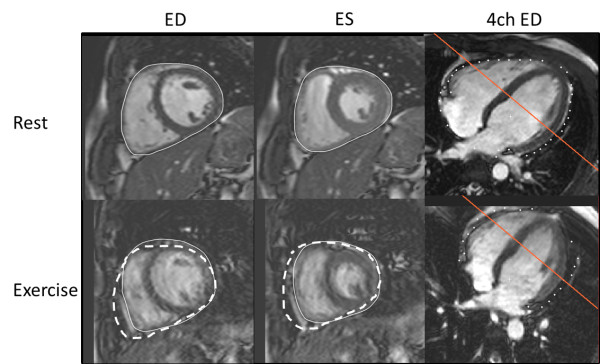
**Mid-ventricular short axis slices in end-diastole (ED) and end-systole (ES) during rest and exercise with the corresponding 4-chamber (4 ch) view to illustrate the location of the slice.** The solid line indicates delineations for total heart volume. In the exercise images, the dashed line shows the total heart volume delineation copied from the corresponding resting image. The right ventricular volume is decreased whereas the left ventricle remains unchanged.

Left ventricular EDV remained unchanged during exercise (186±8 to 185±8 mL, p=0.81) and LVESV decreased from 82±4 to 74±4 mL (p<0.05) (Figure 2A-B). Left ventricular SV increased from 104±4 to 111±5 mL (p<0.05, Figure 2C). For the right ventricle, both RVEDV and RVESV decreased from 203±9 to 185±10 mL for RVEDV and from 100±6 to 77±6 mL for RVESV(p<0.001 for both) (Figure 2D-E) but the increase in right ventricular SV from 104±4 to 108±5 mL was not significant (p=0.18) (Figure 2F). Both left and right ventricular ejection fraction (LVEF and RVEF) increased during exercise from 56±1 to 60±1% and from 52±1 to 59±1% respectively (p<0.01 for both). Cardiac output increased from 6.2±0.3 to 10.4±0.5 L/min (p<0.001) mainly due to the increase in heart rate (Figure 3A-B). Interestingly, total heart volume decreased significantly during exercise by −30±8 mL, (p<0.01) corresponding to a 4±1% decrease of volume (example shown in Figure 4 and in Additional file 4). As expected, LVM was unchanged from rest to exercise (115±6 to 114±6 g, p=0.62).

### Left and right atrio-ventricular plane displacement

Left ventricular AVPD and RVAVPD remained unchanged during exercise. Left ventricular AVPD was 14.6±0.3 mm at rest and 15.3±0.5 mm during exercise (p=0.06) and the RV AVPD was 20.9±0.6 mm for both rest and exercise (p=0.90).

### Longitudinal and radial pumping

**Figure 5:**
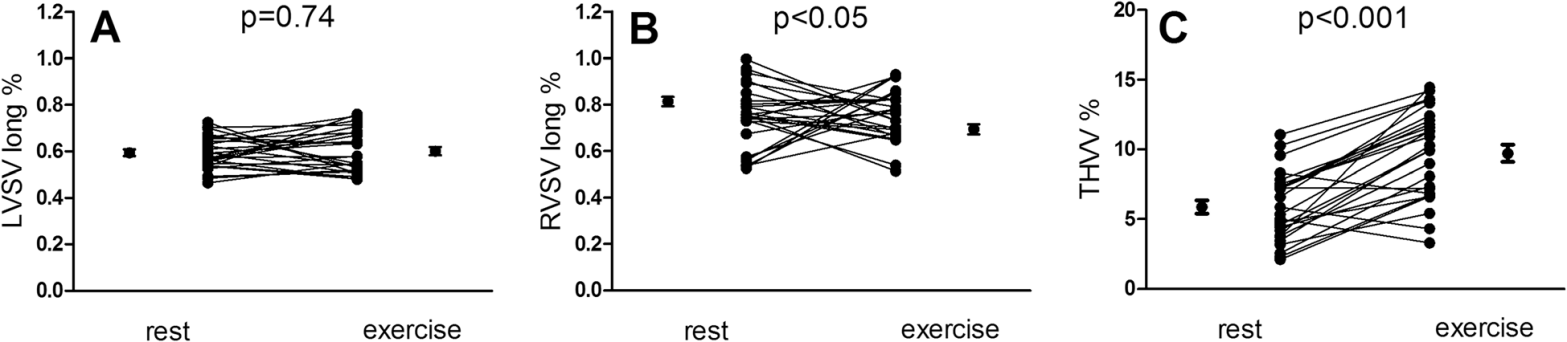
**Longitudinal and radial contribution to stroke volume at rest and exercise.** Left ventricular longitudinal contribution increased **(A)** whereas the right ventricular contribution decreased **(B)**. Total radial contribution calculated as total heart volume variation THVV **(C)** increased significantly indicating an overall increase in radial pumping of the heart during exercise. Error bars denotes mean and standard error of the mean (SEM).

Left ventricular longitudinal contribution to SV (%) remained unchanged at approximately 60% (59±1% at rest and 60±2% at exercise, p=0.74). Right ventricular longitudinal contribution (%) decreased from 81±2 to 75±2% (p<0.05) (Figure 5A-B) due to the decrease in RV end-diastolic volume. Total heart volume variation increased during exercise from 5.9±0.5 to 9.7±0.6% (p<0.001) (Figure 5C).

### Reproducibility of exercise measurements

For the six subjects participating in repeated scans there were no differences in THV, RVEDV or left and right SV between the first and second exercise session with one leg. Left ventricular EDV increased more during the second exercise session when compared to rest; a 5±5% increase during the first session and a 11±4% increase during the second session (p<0.05). When comparing end-expiratory breath hold with end-inspiratory breath hold, only LVEDV differed between sessions. When compared to rest there was a 5±5% increase at end-expiratory breath hold and a 15±7% increase at end-inspiratory breath hold (p<0.05). During exercise using two legs, left and right ventricular EDV did not differ when compared to one-legged exercise. Left ventricular EDV increased by 5±5% and 7±7% respectively (p=0.35), and RVEDV decreased by −4±6% and −2±7% respectively (p=0.17). Right ventricular SV, however, increased more during exercise using two legs. When compared to rest the increase was 3±10% with one leg and 12±9% with two legs.

### Inter-observer variability and validation

Results are presented as mean ±SD. At rest, inter-observer variability for LVM was 9±5 g, EDV −1±4 mL and SV −2±5 mL. During exercise imaging was more difficult and the image quality was lower, which is reflected by a slightly larger variability; LVM 7±10 g, EDV −3±16 mL and SV 0±9 mL.

## Discussion

The present study has shown that the total heart volume decreases in healthy normal subjects during moderate exercise in the supine position. This decrease is caused by reduced right atrial and ventricular volumes whilst left atrial and ventricular volumes remain unchanged during exercise. With regards to pumping function there is an increase in outer volume changes during exercise and thus, an increased radial contribution to stroke volume. AV-plane movement is unchanged during exercise but a smaller short-axis area of the right ventricle causes the lower longitudinal contribution to RVSV. Left ventricular longitudinal contribution to SV is unchanged during exercise. Total left and right ventricular SV were only slightly increased (LVSV) or unchanged (RVSV) during supine exercise and the increase in cardiac output is best explained by the rise in heart rate.

### Ventricular volumes and stroke volume

The inconsistent results of previous studies [4-7,11,12,14,26] may be explained by differences in imaging modalities and, perhaps most important, body position. The results of this study are in line with other studies of supine exercise showing unchanged LVEDV [5,9,10,27,28]. The significant decrease in RVEDV differs from previous studies of RV volumes during supine exercise using radionuclide ventriculography [13] and CMR [14,28] where RVEDV remained unchanged during moderate intensity exercise (mean HR in these studies were 112,120 and 100 bpm respectively). However, in line with our results the study by Mols et al. [13] used radionuclide ventriculography and showed a decreased RVEDV at workloads at a HR of 127 bpm and above. The differences between the present study and previous CMR studies may be explained by differences in exercise protocol where we acquired breath-hold images during leg exercise whilst Holverda et al. [28] used non-breath hold imaging and Roest et al. [14] allowed the subjects to rest for the short period of image acquisition. Free-breathing may decrease image quality and rest during image acquisition will allow HR to decrease making interpretation of results more difficult.

In line with studies by Bevegård et al. [29] the present study showed that CO increased significantly due to increased HR whilst SV only increased by 8%. During the early stages of exercise in healthy subjects, the increase in HR is primarily caused by a decreased parasympathetic tone whereas the sympathetic effects are not seen until later stages [30]. As the exercise bouts of the study were short, the increased HR with only a small increase in SV is likely caused by parasympathetic withdrawal. Furthermore, the increased venous return caused by the supine position lead to maximal filling of the ventricles already at rest, which would explain the discrepancy between our study and exercise studies performed in the upright position. Our study would then be more representative of exercise in the supine position such as swimming or perhaps in micro gravitational environments such as space flight.

### Longitudinal and radial contribution to stroke volume

In contrast to a previous study of upright exercise on an ergometer cycle where the left ventricular valve displacement was significantly increased during exercise [20], our results showed unchanged LV AVPD and longitudinal contribution to LVSV. Right ventricular valve displacement (RV AVPD) remained unchanged but together with the decreased volume of the right ventricle, the right ventricular longitudinal contribution to SV was significantly decreased. Furthermore, total cardiac pumping became significantly more radial during exercise as shown by the increased THVV when exercising both with one and two legs, as well as during end-expiratory and end-inspiratory breath hold. This is in contrast to a hypothesis previously suggested by our group [22] where we expected cardiac longitudinal pumping to increase and radial pumping to decrease. Increased radial pumping as seen in the present study may theoretically increase the amount of energy spent on moving surrounding tissues and thus decrease the energy efficiency of the heart. However, for the left ventricle, Riordan and Kovács [31] showed that radial pumping may actually be important for diastolic suction during the rapid filling phase. Exercise requires rapid mass transfer from the atria to the ventricle, and it is possible that the increased radial pumping seen in the right ventricle may actually improve cardiac pumping efficiency due to enhanced diastolic suction.

It is possible that our findings of increased THVV only relates to exercise in the supine position such as swimming, and it would be of interest to perform similar studies during upright exercise.

### Reproducibility of exercise measurements

Ventricular volumes and THV were reproducible between the first and second exercise session, and also when imaging was performed at end-inspiratory breath hold as well as during exercise with two legs. The differences seen in LVEDV between the first and second exercise session with one leg as well as between end-expiratory and end-inspiratory breath hold is probably best explained by individual variations that are more distinguishable in the small population. As shown in Figure 2 there is some variability between individuals for all variables and when only assessing six subjects results may fall out as statistically significant although not physiologically relevant.

### Clinical implication

Heart failure is a complex syndrome and diagnosis can be especially challenging at early stages. Cardiac MR during physical exercise may become useful for assessing patients with normal ejection fraction and suspected heart failure to investigate if cardiac function and filling are affected during low and medium intensity exercise. Furthermore, exercise CMR may also be used to asses patients with congenital heart disease such as Tetralogy of Fallot before and after surgery.

### Limitations

Exercise heart rate in our healthy volunteers only increased by approximately 40 bpm over resting HR and it is possible that a higher exercise HR may yield different results. The study population included to test for reproducibility of exercise measurements was small (n=6) and the results of the statistical tests of this subpopulation on reproducibility should be interpreted with caution. Furthermore, the study was performed in the supine position limiting the interpretation of our results to supine exercise such as swimming, but it may also be applicable for conditions of microgravity, such as space flight.

## Conclusions

Moderate intensity exercise in the supine position significantly decreases the total heart volume. This is due to decreases in right atrial and ventricular volumes at end-diastole whilst the LVEDV remains unchanged. The contribution of longitudinal pumping to stroke volume is unchanged in the left ventricle but decreased in the right ventricle in exchange for an increase in radial pumping. In contrast to previous belief, THVV and consequently radial pumping increases which may improve diastolic suction of the ventricles.

## Competing interests

The authors declared that they have no competing interest.

## Authors’ contributions

KSE: Conception of study, data inclusion and analysis, interpretation of data, drafting and revising the manuscript. RJ: Data inclusion and critical revision of the manuscript. PMA: Data inclusion and analysis, critical revision of the manuscript. MC: Conception of study, data inclusion and critical revision of the manuscript. BS: Conception of study, construction of MR ergometer, critical revision of manuscript. HA: Conception of study, critical revision of manuscript. All authors read and approved the final manuscript.

## Supplementary Material

Additional file 1 Short-axis image of a healthy heart during exercise at a heart rate of 108 bpm. Click here for file

Additional file 2 Two-chamber long-axis view of a healthy heart during exercise at a heart rate of 102 bpm. Click here for file

Additional file 3 Three-chamber long-axis view of a healthy heart during exercise at a heart rate of 117 bpm. Click here for file

Additional file 4 Four-chamber long-axis view of a healthy heart during exercise at a heart rate of 124 bpm. Click here for file

## References

[B1] Steding K Engblom H Buhre T Carlsson M Mosen H Wohlfart B Arheden H Relation between cardiac dimensions and peak oxygen uptake J Cardiovasc Magn Reson 2010 15 1 8 20122149 10.1186/1532-429X-12-8 PMC2825210

[B2] Henschen ES Skiddlauf und skidwettlauf. Eine medizinische sportstudie. Mitt. Med. klin 1899 Upsala: Jena Fischer Verlag

[B3] Widmaier E Raff H Strang K Human Physiology. The Mechanisms of Body Function 2008 11 New York: McGRaw-Hill

[B4] Bar-Shlomo BZ Druck MN Morch JE Jablonsky G Hilton JD Feiglin DH McLaughlin PR Left ventricular function in trained and untrained healthy subjects Circulation 1982 15 3 484 8 7055870 10.1161/01.cir.65.3.484

[B5] Crawford MH White DH Amon KW Echocardiographic evaluation of left ventricular size and performance during handgrip and supine and upright bicycle exercise Circulation 1979 15 6 1188 96 436212 10.1161/01.cir.59.6.1188

[B6] Fagard R Van den Broeke C Amery A Left ventricular dynamics during exercise in elite marathon runners J Am Coll Cardiol 1989 15 1 112 8 2738255 10.1016/0735-1097(89)90060-0

[B7] Henriksen E Sundstedt M Hedberg P Left ventricular end-diastolic geometrical adjustments during exercise in endurance athletes Clin Physiol Funct Imaging 2008 15 2 76 80 18076659 10.1111/j.1475-097X.2007.00768.x

[B8] Rerych SK Scholz PM Sabiston DC Jr Jones RH Effects of exercise training on left ventricular function in normal subjects: a longitudinal study by radionuclide angiography Am J Cardiol 1980 15 2 244 52 7355734 10.1016/0002-9149(80)90642-6

[B9] Stein RA Michielli D Diamond J Horwitz B Krasnow N The cardiac response to exercise training: echocardiographic analysis at rest and during exercise Am J Cardiol 1980 15 2 219 25 7405835 10.1016/0002-9149(80)90061-2

[B10] Stein RA Michielli D Fox EL Krasnow N Continuous ventricular dimensions in man during supine exercise and recovery. An echocardiographic study Am J Cardiol 1978 15 4 655 60 645568 10.1016/0002-9149(78)90813-5

[B11] Sundstedt M Hedberg P Jonason T Ringqvist I Brodin LA Henriksen E Left ventricular volumes during exercise in endurance athletes assessed by contrast echocardiography Acta Physiol Scand 2004 15 1 45 51 15329056 10.1111/j.1365-201X.2004.01304.x

[B12] Sundstedt M Jonason T Ahren T Damm S Wesslen L Henriksen E Left ventricular volume changes during supine exercise in young endurance athletes Acta Physiol Scand 2003 15 4 467 72 12648164 10.1046/j.1365-201X.2003.01098.x

[B13] Mols P Huynh CH Naeije N Ham HR Volumetric response of right ventricle during progressive supine exercise in men Am J Physiol 1991 15 3 Pt 2 H751 4 1887922 10.1152/ajpheart.1991.261.3.H751

[B14] Roest AA Kunz P Lamb HJ Helbing WA van der Wall EE De Roos A Biventricular response to supine physical exercise in young adults assessed with ultrafast magnetic resonance imaging Am J Cardiol 2001 15 5 601 5 11230846 10.1016/s0002-9149(00)01438-7

[B15] Carlsson M Ugander M Heiberg E Arheden H The quantitative relationship between longitudinal and radial function in left, right, and total heart pumping in humans Am J Physiol Heart Circ Physiol 2007 15 1 H636 44 17307988 10.1152/ajpheart.01376.2006

[B16] Lundbäck S Cardiac pumping and function of the ventricular septum Acta Physiol Scand 1986 15 8 101 3464160

[B17] Steding-Ehrenborg K Carlsson M Stephensen SS Arheden H Atrial aspiration from pulmonary and caval veins is caused by ventricular contraction and secures 70% of the total stroke volume independent of resting heart rate and heart size Clin Physiol Funct Imaging 2013 15 3 233 40 23522018 10.1111/cpf.12020

[B18] Waters EA Bowman AW Kovacs SJ MRI-determined left ventricular “crescent effect”: a consequence of the slight deviation of contents of the pericardial sack from the constant-volume state Am J Physiol Heart Circ Physiol 2005 15 2 H848 53 15486032 10.1152/ajpheart.00744.2004

[B19] Carlhall CJ Lindstrom L Wranne B Nylander E Atrioventricular plane displacement correlates closely to circulatory dimensions but not to ejection fraction in normal young subjects Clinical physiology 2001 15 5 621 8 11576164 10.1046/j.1365-2281.2001.00356.x

[B20] Sundstedt M Hedberg P Henriksen E Mitral annular excursion during exercise in endurance athletes Clin Physiol Funct Imaging 2008 15 1 27 31 18171401 10.1111/j.1475-097X.2007.00769.x

[B21] Brecher GA Cardiac variations in venous return studied with a new bristle flowmeter Am J Physiol 1954 15 3 423 30 13138732 10.1152/ajplegacy.1954.176.3.423

[B22] Carlsson M Cain P Holmqvist C Stahlberg F Lundback S Arheden H Total heart volume variation thoughout the cardiac cycle in humans Am J Physiol Heart Circ Physiol 2004 15 243 50 10.1152/ajpheart.01125.2003 15016625

[B23] Gauer OH Volume changes of the left ventricle during blood pooling and exercise in the intact animal; their effects on left ventricular performance Physiol Rev 1955 15 1 143 55 14356928 10.1152/physrev.1955.35.1.143

[B24] Heiberg E Sjogren J Ugander M Carlsson M Engblom H Arheden H Design and validation of Segment - freely available software for cardiovascular image analysis BMC Med Imaging 2010 15 1 1 20064248 10.1186/1471-2342-10-1 PMC2822815

[B25] Carlsson M Ugander M Mosen H Buhre T Arheden H Atrioventricular plane displacement is the major contributor to left ventricular pumping in healthy adults, athletes, and patients with dilated cardiomyopathy Am J Physiol Heart Circ Physiol 2007 15 3 H1452 9 17098822 10.1152/ajpheart.01148.2006

[B26] Widmaier E Raff H Strang K Mechanical events of the cardiac cycle Human Physiology - The Mechanisms for Body Function 1996 10 New York: McGraw-Hill Higher Education 404 5

[B27] Schairer JR Stein PD Keteyian S Fedel F Ehrman J Alam M Henry JW Shaw T Left ventricular response to submaximal exercise in endurance-trained athletes and sedentary adults Am J Cardiol 1992 15 9 930 3 1529949 10.1016/0002-9149(92)90741-g

[B28] Holverda S Gan CT Marcus JT Postmus PE Boonstra A Vonk-Noordegraaf A Impaired stroke volume response to exercise in pulmonary arterial hypertension J Am Coll Cardiol 2006 15 8 1732 3 16631018 10.1016/j.jacc.2006.01.048

[B29] Bevegard S Studies on the regulation of the circulation in man. With special reference to the stroke volume and the effect of muscular work, body position and artificially induced variations of the heart rate Acta physiologica Scandinavica Supplementum 1962 15 200 1 36 13967907

[B30] Chaitman BR Should early acceleration of heart rate during exercise be used to risk stratify patients with suspected or established coronary artery disease? Circulation 2007 15 4 430 1 17261670 10.1161/CIRCULATIONAHA.106.676882

[B31] Riordan MM Kovacs SJ Relationship of pulmonary vein flow to left ventricular short-axis epicardial displacement in diastole: model-based prediction with in vivo validation Am J Physiol Heart Circ Physiol 2006 15 3 H1210 5 16603684 10.1152/ajpheart.01339.2005

